# A Comparability of Renal Length and Volume Measurements in MRI and Ultrasound in Children

**DOI:** 10.3389/fped.2021.778079

**Published:** 2021-12-08

**Authors:** Dominik Świętoń, Weronika Bernard, Małgorzata Grzywińska, Piotr Czarniak, Agata Durawa, Mariusz Kaszubowski, Maciej Piskunowicz, Edyta Szurowska

**Affiliations:** ^1^Second Radiology Department, Medical University of Gdansk, Gdańsk, Poland; ^2^Faculty of Medicine, Medical University of Gdansk, Gdańsk, Poland; ^3^Department of Human Physiology, Medical University of Gdansk, Gdansk, Poland; ^4^Department of Pediatrics, Nephrology and Hypertension, Medical University of Gdansk, Gdańsk, Poland; ^5^Department of Statistics and Econometrics, Faculty of Management and Economics, Gdansk University of Technology, Gdansk, Poland; ^6^First Department of Radiology, Medical University of Gdansk, Gdańsk, Poland

**Keywords:** renal volume, renal length, kidney MRI, kidney ultrasound, renal measurements, renal MRI and US

## Abstract

**Introduction:** Despite the significant increase in use of magnetic resonance imaging (MRI) in children, there is still a lack of normal reference values of renal size in this method and reference values are being interpolated from the ultrasound (US) studies. The study provides comparative analysis of agreement in renal length and volume measurements between MRI and ultrasound.

**Materials and Methods:** Ninety-three children with a mean age of 8.0 ± 6.0 years, who had undergone both renal US and MRI exams, were included in the study. Participants were divided into three subgroups; each kidney was considered separately.

Group 1 included 106 kidneys without any anomalies. Group 2 comprised 48 kidneys with a dilated collecting system. Group 3 included 32 kidneys with a duplicated collecting system. Measurements were taken in three dimensions, and renal volume was calculated from the ellipsoid formula.

**Results:** We found no significant difference between US and MRI measurements in Group 1 and Group 2. In Group 3, the difference between measurements in both imaging methods was significant. The mean difference varied from 0.05% in Group 1, 2.95% in Group 2, to 4.99% in Group 3.

**Conclusion:** The US and MRI are comparable methods in renal size measurements. The interpolation of sonographic renal length and volume reference values to the MRI in the pediatric population is justified, as there is a strong agreement between both methods. Both methods can be used interchangeably for following up of the renal size changes in the pediatric population.

## Introduction

The renal volume is a clinically important parameter, giving indirect information about the renal parenchymal volume and renal functional reserve. It is an important imaging parameter for monitoring patients with chronic kidney disease (1–3). The first-choice imaging modality in children with nephrological and urological pathologies is the ultrasound (US). The reference values for sonographic measurements of renal size are accessible for a healthy pediatric population (4–11). The next popular and rapidly developing imaging method is the magnetic resonance imaging (MRI). Unlike for US, the reference values of renal size for the MRI are still unavailable due to limited access to this imaging modality, and secondly due to ethical issues. The normal values of renal size for the MRI in the pediatric population are mainly available on dedicated websites and demonstrate norms adopted from US reference values, whereas in adults, many papers presenting renal size reference values in a healthy population, and a variety of kidney diseases, are available (12).

There are a limited number of studies comparing the accuracy of US and MRI in renal size assessment. However, all are based on the exams in adults or *in vitro*, using phantoms or animals (13–15). Pediatric patients are specific due to changes in the size of most organs with age, and the different spectra of affecting diseases, compared with adults. We observe a significant increase in magnetic resonance urography (MRU) use in the pediatric population, as a radiation-free modality, giving comprehensive information about renal morphology and function (16–18). Therefore, normal values of renal size for MRI/MRU are necessary, as well as information about the comparability of both methods. Instead of creating separate renal growth charts for the MRI, we compared the coherence of both methods in renal length and volume assessment in the pediatric population.

Additionally, we analyzed and compared both methods in the assessment of duplex kidneys and kidneys with dilatation of the collecting system. The last-mentioned group is very important for pediatric nephrologists and urologists, as the kidney volume can correlate with the severity of renal obstruction (3).

## Materials and Methods

### Study Group

A retrospective analysis of renal MRI examinations was performed in 142 consecutive patients examined from January 2013 to September 2019. From this group, we selected 93 children with both MRI and US exams performed with the interval time between examinations not exceeding 6 months. Due to distinct growth of infants, the time interval was acceptable when it was under 3 months. In our study group, the mean value of the time interval was 1 month for the infants under 12 months (18 patients) and 2 months in children between 12 and 24 months of life (9 patients). The mean age of children was 8.0 ± 6.0 years, range: 3 months-18 years.

Children underwent MRI examinations due to uropathies, mostly obstructive, or arterial hypertension. The results were divided into subgroups, and each kidney was treated as a separate kidney unit (KU); in total, 186 KUs were analyzed. If patients had normal and abnormal kidney, both were classified separately to dedicated groups.

Group 1 included 59 patients with at least one normal kidney without a dilatation of collecting system or other anomalies, in 12 patients only one kidney met the inclusion criteria, and finally 106 KUs were enrolled for further analysis. Group 2 consisted of 48 KUs with a dilated collecting system. Group 3 comprised 32 KUs with a duplicated collecting system. In all children, kidney measurements were taken in three dimensions in US and MRI reports. We used the ellipsoid formula for volume calculation for each method:


$$\begin{array}{l} \text{Vol}=\text{ length}\cdot \text{width}\cdot \text{depth}\cdot 0.523\left[\text{ml}\right] \end{array}$$

The statistical analysis of the correlation between the US and MRI measurements was performed separately for each group, both including the division between left and right kidneys, and regardless of the side.

We used the following formulas to determine the relative differences between kidney length and volume assessed in US vs. MRI.

For renal length
Relative difference in renal length =
$\frac{Length\;in\;MRI-length\;in\;US}{length\;in\;MRI}\cdot \;100\%$.
a)For renal volume
Relative difference in renal volume =
$\frac{volume\;in\;MRI-volume\;in\;US}{volume\;in\;MRI}\cdot \;100\%$.

### MRI Imaging

The examinations were performed on a Philips Achieva 3.0T TX magnetic resonance scanner (Philips Healthcare, Best, The Netherlands) with a 16-channel coil dedicated to abdominal examinations. Children under 6 years old were examined in general anesthesia.

**Figure 1:**
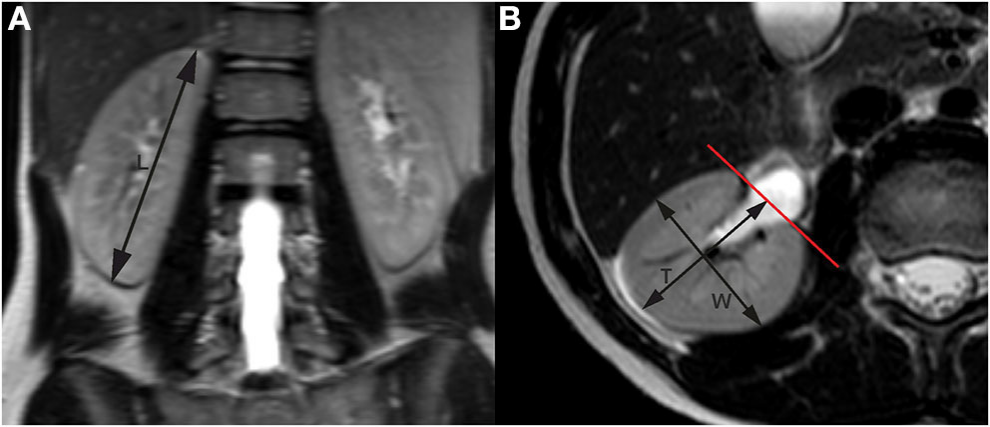
Renal measurements by MRI. **(A)** Longitudinal measurements in coronal plane; **(B)** transverse section at the level of the renal hilum. MRI, magnetic resonance imaging.

Kidney measurements were taken in sequences based on T2-weighted images, the longitudinal diameter in the coronal plane (VISTA_COR_Sense), while depth and width were taken from transverse scans (T2W_TSE_Tra_HR). The coronal view was corrected in MPR projection to get the longest longitudinal dimension of each kidney. The length of kidneys was taken as a distance between both poles, and the depth and width were taken in transverse scans at the level of the hilum, both orthogonally (Figure 1).

All exams were assessed by the same radiologist (D.S.) with 10 years of experience in MRI.

### US Imaging

**Figure 2:**
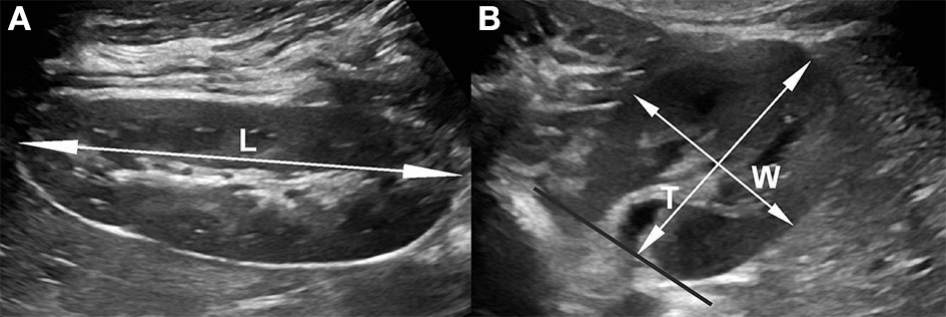
Renal measurements by US B mode, with convex probe 1-6MHz. **(A)** Longitudinal section, assessed as the maximum renal length between upper and lower pole; **(B)** transverse section, two measurements taken orthogonally at the level of the renal hilum. US, ultrasound.

The US examinations were performed by two physicians experienced in renal US imaging (P.C.—pediatric nephrologist with 25 years of experience, D.S.—pediatric radiologist with 15 years experience) on GE Voluson S8 (GE Medical Systems, Milwaukee, WI, USA), and Philips Epiq 5 (Philips Ultrasound, Bothell, WA, USA), using linear, high-frequency 12–18-MHz probes and convex 1–6-MHz probes. Measurements were taken in a prone position in children under 15 years of age, and in both right and left decubitus positions in older children (Figure 2). The length of kidneys was taken as a distance between both poles; the depth and width were taken on transverse scans at the level of the hilum, perpendicularly to the long axis of the kidney and orthogonally to each other.

### Statistics

Data collected from MRI and US examinations were both included in the statistical analysis.

Both methods were compared in two ways: considering the left and right sides and without differentiation between sides. The correlation between MRI and US measurements was calculated with Spearman's rank test. The compatibility between length and volume measurements in both methods is presented with a Bland–Altman plot. For a graphical presentation of the differences between both methods, we used the difference between measurements in MRI and US (valueMR—valueUS) and mean values of the difference [(value MR + value US)/2]. The one-sample-test shows which group does not have a statistically significant difference between the two measurements.

To reflect bias in the scatter plot, we used the test for linear trend—linear regression. To analyze the difference between measurements, we used the Mann–Whitney *U*-test. A *p*-value < 0.05 was statistically significant. Statistical analysis was performed with IBM SPSS Statistic 25 software.

## Results

**Figure 3:**
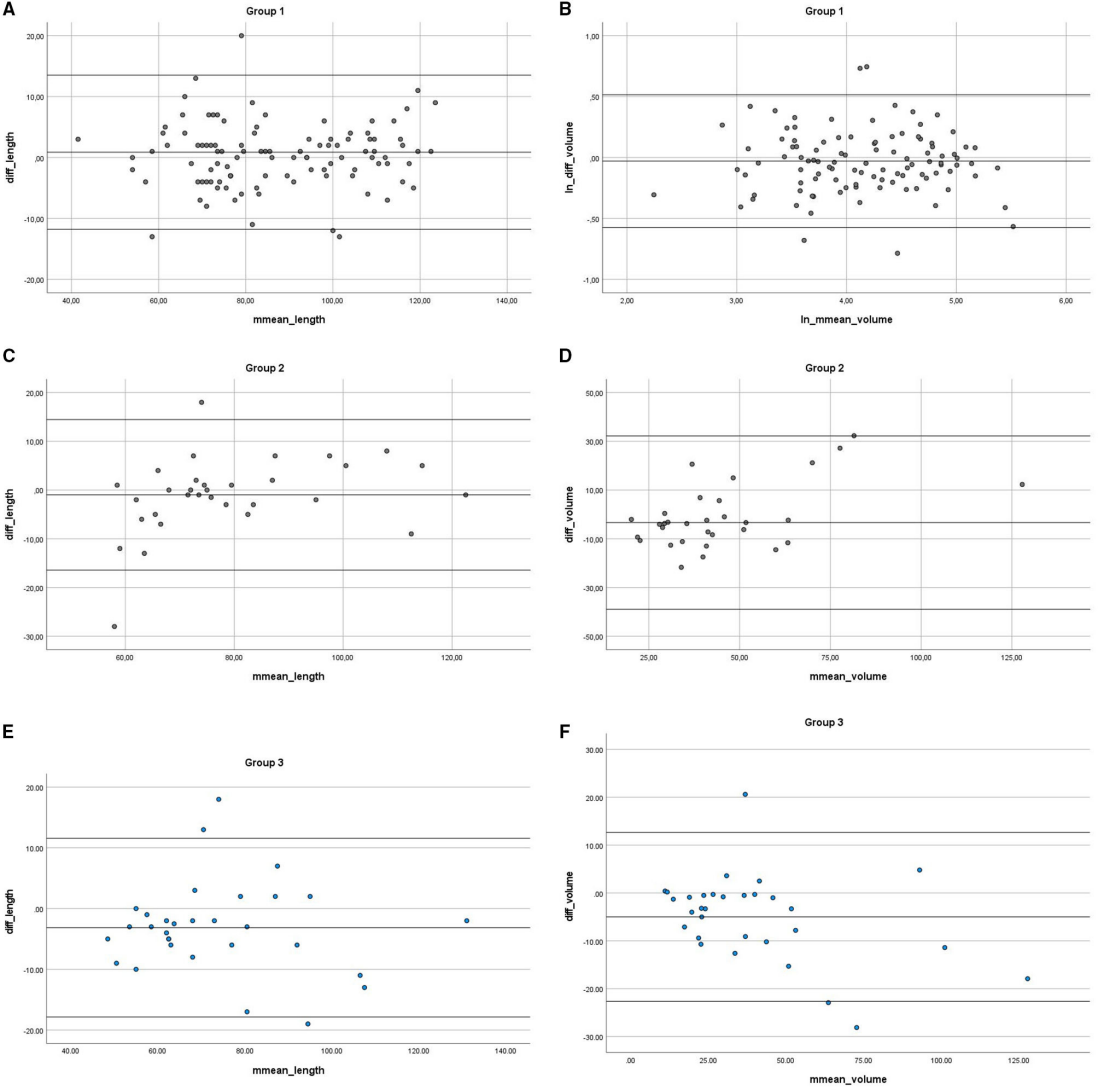
Bland-Altman plot of the difference in measurements of renal length and volume in MRI and US **(A,B)** for group 1, **(C,D)** for group 2 and **(E,F)** for group 3. MRI, magnetic resonance imaging; US, ultrasound.

The studied groups 1 and 2 revealed no significant difference in assessment of renal length (*p* = 0.162 and *p* = 0.485, respectively) and volume (*p* = 0.283 and *p* = 0.304, respectively). The Bland–Altman plots used in the analysis of agreement between both methods are presented in the graphs (Figures 3A–D). The linear regression output showed no significant values, indicating no trend—both methods are equivalent. We found the trend of US to overestimate the measurements compared with MRI in both groups, but not statistically significant. The mean percentage difference for the length in group 1 was 0.05 and 2.95% in group 2.

In Group 3, we obtained a statistically significant difference between the two methods (*p*_length_ =0.027, *p*_volume_ =0.004) (Figures 3E,F), where mean differences were significant for length and volume (mean_difference_length_ = −3.145; mean_difference = −4.993). The results show US slightly overestimating, when compared to MRI results (skewness_length_ = 0.554; kurtosis_length_ = 1.798, median_length_ = −3.000), with the mean percentage difference in length measurement of 4.99%.

## Discussion

Our study confirmed that size measurements of healthy kidneys are comparable in both imaging methods (*p* < 0.162). In US, we found slight trends to overestimate the values, but statistically not significant. Studies based on adults or phantoms suggest an underestimation of kidney volume in US, which was not found in our study group (12, 14). Our results showed a lower disproportion between results in both methods in comparison to papers based on the adult population. In our opinion, the assessment of kidneys in children with US is more convenient, which can interfere with the accuracy of measurements, especially when examined in prone position. In our study, the disproportion in length and volume between the methods was not statistically significant for normal kidneys; the difference did not exceed 0.05% while reaching 5% in duplex kidneys. In our opinion, the enlargement of duplex kidneys and their position can interfere with precise renal length measurements. In children, the distance between the kidney and probe surface is shorter and sometimes it is difficult to visualize the full length of the kidney on one scan. We did not find any significant influence of kidney side location on measurement results in any method.

Several publications suggest the disk-summation method in MRI as the most precise way of renal volume assessment. However, we chose the method based on three-dimensional measurements of renal volume followed by ellipsoid formula calculation (12). Hereby, we could compare volumes assessed in the same way in both imaging methods, as the disk-summation method is not accessible in 2D mode sonography. Secondly, the assessment of the volume based on an ellipsoid formula is commonly accepted and is easy for everyday radiological practice, as well in US as in MRI. However, it should be noted that the ellipsoid formula of renal volume calculation is burdened with an error up to 18%, when compared with the water displacement method *ex vivo* (12). Therefore, the next prospective studies are necessary to compare MRI an US in volume calculation using ellipsoid formula and disk-summation method, but 3D US will be necessary.

Full compliance between both methods was found in measurements of kidneys with dilatation of the collecting system (*p* < 0.05). The renal length and volume are valuable clinical parameters in the follow-up of pediatric patients with congenital hydronephrosis, following kidney transplantation, in arterial hypertension, in monitoring polycystic kidney disease, and in differentiation between acute and chronic kidney injury. Thus, the information that US and MRI are comparable methods in measurement of renal size, even in kidneys with uropathies, is vital (2, 3, 19, 20).

MRI provides high-resolution images of the kidneys and collecting systems, as well as functional parameters. The use of this modality for determination of urological ambiguities after US screening is becoming more frequent (17, 18, 21). Along with the increase in the use of MRU, new potential utilities of the method appear. Therefore, the conclusions of our study seem to be vital for clinical use.

To our best knowledge, there are still no reference values of kidney size assessed in MRI for the pediatric population. Our findings justify interpolation of sonographic renal size reference values to the MRI.

## Conclusions

The US and MRI are comparable methods in renal size measurements. The interpolation of sonographic renal length and volume reference values to the MRI in the pediatric population is justified, as there is a strong agreement between both methods. Both methods can be used interchangeably for follow-up of the renal size changes in the pediatric population.

## Data Availability Statement

The raw data supporting the conclusions of this article will be made available by the authors, without undue reservation.

## Ethics Statement

The studies involving human participants were reviewed and approved by Independent Bioethics Committee for Scientific Research at Medical University of Gdańsk. Written informed consent from the participants' legal guardian/next of kin was not required to participate in this study in accordance with the national legislation and the institutional requirements.

## Author Contributions

Material preparation, data collection, and analysis were performed by DŚ, WB, and MG. The first draft of the manuscript was written by DŚ and DŚ, WB, MG, PC, AD, MK, MP, and ES on previous versions of the manuscript. All authors contributed to the study conception, design, read, and approved the final manuscript.

## Conflict of Interest

The authors declare that the research was conducted in the absence of any commercial or financial relationships that could be construed as a potential conflict of interest.

## Publisher's Note

All claims expressed in this article are solely those of the authors and do not necessarily represent those of their affiliated organizations, or those of the publisher, the editors and the reviewers. Any product that may be evaluated in this article, or claim that may be made by its manufacturer, is not guaranteed or endorsed by the publisher.

## Abbreviations

MRI: magnetic resonance imaging
MRU: magnetic resonance urography
US: ultrasound
Vol: volume.
